# The usefulness of C-reactive protein in predicting malaria parasitemia in a sub-Saharan African region

**DOI:** 10.1371/journal.pone.0201693

**Published:** 2018-08-06

**Authors:** Bismark Osei Sarfo, Andreas Hahn, Norbert Georg Schwarz, Anna Jaeger, Nimako Sarpong, Florian Marks, Yaw Adu-Sarkodie, Thalea Tamminga, Juergen May

**Affiliations:** 1 Bernhard Nocht Institute for Tropical Medicine, Hamburg, Germany; 2 Institute for Microbiology, Charité Berlin University of Medicine, Berlin, Germany; 3 Kumasi Centre for Collaborative Research, Kumasi, Ghana; 4 International Vaccine Institute, Seoul, South Korea; 5 Kwame Nkrumah University of Science and Technology, Kumasi, Ghana; 6 Robert Koch Institute, Berlin, Germany; 7 University Medical Centre, Hamburg Eppendorf, Germany; 8 German Center for Infectious Disease Research (DZIF), Hamburg-Borstel-Lübeck, Germany; Universidade Nova de Lisboa Instituto de Higiene e Medicina Tropical, PORTUGAL

## Abstract

**Background:**

Malaria remains a leading cause of childhood mortality in sub-Saharan Africa. Identifying patients who are at risk for severe manifestations at presentation still remains challenging. This study examines whether a semi-quantitative test on C-Reactive Protein (CRP) could be useful for rapidly predicting the presence or absence of malarial parasitemia in febrile children.

**Method:**

Data were collected from children with fever or a history of fever at the Agogo Presbyterian Hospital in the Ashanti Region of Ghana. Haematological measurements, microscopic detection of plasmodium species and semi-quantitative CRP measurements with a membrane–based immunoassay for whole blood were performed. CRP was classified as positive when the measured level was ≥ 10 mg/l.

**Results:**

During 548 visits, thick blood film results could be obtained from 541 patients, 270 (49.3%) yielded parasitemia with *Plasmodium* spp. Whereas malaria parasites were detected in only a few patients (7.1%) with normal CRP levels (< 10mg/l), more than a half of patients with an increased CRP concentration (≥ 10 mg/l) were parasite positive (OR 14.5 [CI 4.4–47.6], p<0.001). Patients with increased CRP levels had more than an eight-fold likelihood for parasitemia after correction for other parameters (adjusted OR 8.7 [CI 2.5–30.5], p<0.001). Sensitivity, specificity as well as positive predictive and negative predictive values of CRP for malaria were 99.3% (CI 96.2%-100%), 9.2% (CI 6.4%-12.8%), 31.7% (CI 27.4%-36.1%) and 97.0% (CI 84.2%-99.9%), respectively.

**Conclusion:**

The semi-quantitative method of measuring CRP is cheap, rapid and easy to perform but not useful in predicting parasitemia and malaria. However, due to its high negative predictive value, it could have a role in identifying those patients unlikely to be presenting with clinical malaria.

## Background

*Plasmodium falciparum* malaria is among the leading causes of childhood mortality in most sub-Saharan African countries [1]. In 2017, the World Health Organization (WHO) estimated that among the 445,000 deaths attributable to malaria worldwide, most occurred in African children less than five years old [2]. The majority of these children have to rely on underresourced health facilities, especially in terms of laboratory investigations and personnel.

The case definition of malaria requires at least an increased body temperature and the presence of asexual stages of malaria parasites. However, as a result of the intermittent nature of malaria fever [3], some malaria cases may present afebrile even in patients with high parasite density in areas of high endemicity [4,5]. Fever due to other viral or bacterial infections might mislead to the diagnosis of malaria in patients with parasitemia. Symptoms, which have been described in malaria such as headache, fatigue, abdominal discomfort, joint aches also do occur in viral or bacterial infections [6]. On the other side, clinical manifestations of severe malaria, which include cerebral manifestations and severe anaemia, may overlap with those of other severe febrile illnesses in endemic countries [7], thereby making it difficult to determine which patients really have malaria at presentation. Rapid identification of patients who may not have malaria, or have another diagnosis along with malaria, is crucial to institute timely therapy and thereby decrease the fatality rate.

In highly endemic regions, parasitemia can be a common finding and clinical manifestations can be due to causes other than malarial infection. The WHO therefore recommends that malaria microscopy or rapid diagnostic test (RDT) be carried out in suspected malaria cases [6]. In a context where these may not be available, other biomarkers could also improve an early specific diagnosis of clinical malaria and enhance the distinction between malaria and other diseases.

Blood glucose, lactate concentration and base excess have long been used in assessing severe malaria and its complications. Biomarkers related to the immune activation pathways such as procalcitonin, angiopoeitin and von Willebrand Factor (vWF) have recently been studied [8,9]. Though these tests may be useful in the diagnosis and prognosis of malaria, they were not systematically tested for this purpose in endemic regions. Moreover, time and costs of these tests would make them inappropriate for routine use in most African settings.

C-Reactive Protein (CRP) has also been studied lately in patients with malaria in Africa and other parts of the world [4,10,11]. CRP is an acute-phase reactant, whose plasma concentration increases during inflammatory disorders [12,13]. It is released during malaria [4,10,11,14] and other infections [15,16,17]. Beside activation of the complement pathway, CRP exhibits an important phagocytic function, binding infected erythrocytes and helping in their clearance [18]. It is also valuable for assessing the severity of malaria and as prognostic tool in the follow-up response to treatments [14].

CRP can be measured qualitatively, semi-quantitatively and quantitatively. The qualitative method measures the absence or presence of a certain level of CRP in a serum sample [19]. The semi-quantitative method measures an approximate concentration of CRP within a serum sample. The quantitative method is more complex and expensive, requiring more time to perform (15–30 minutes) [15]. This method is widely used in developed countries, providing relatively rapid, highly sensitive and specific results. The qualitative and semi-quantitative methods are simpler, less expensive and require less skill to perform. They can be used as point-of-care bed-side (POC) tests, not necessarily requiring a standard laboratory, therefore making these methods more feasible in many areas of sub-Saharan Africa.

The aim of this study was to evaluate the value of a semi-quantitative CRP test to predict clinical malaria among febrile children in a highly endemic area of Ghana.

## Materials and methods

### Study design

Patients were recruited between January and October 2010. Data were collected from children aged up to 15 years old with fever or a history of fever during the 24 hours preceding the consultation. Haematological measurements, semi-quantitative CRP measurements and microscopic *Plasmodium* detection were performed and analysed. All samples were collected at the Agogo Presbyterian Hospital in the Asante Akyem North district in Ghana. The predominant malaria parasite in this region is *P*. *falciparum* [20] with an estimated entomological inoculation rate (EIR) of > 400 per person per year [21]. All statistical analyses were performed using StataMP 12 (StataCorp., Texas, USA).

Body temperatures were recorded for each child, and fever was defined as an axillary temperature > 37.5°C. Venous blood was drawn using Vacutainer tubes (Becton Dickinson, Oxford, UK) containing EDTA as an anti-coagulant. A blood smear was immediately made and later stained with Giemsa. The full blood count (FBC) analysis for each participant`s sample was analysed using a Sysmex^R^ KX-21N Haematological Analyzer (Sysmex Corporation, Kobe, Japan).

### Consent and ethical approval

The study was conducted in accordance with the ethical principles of the Declaration of Helsinki. Ethical approval was obtained from the Ethics committees of the School of Medical Sciences, Kwame Nkrumah University of Science and Technology (KNUST), Kumasi.

Written informed consents of all study participants were obtained from mothers or guardians after been informed about the aim of the study in presence of a witness.

### Parasitology

*Plasmodium* species and counts (number of parasites per 200 leukocytes) were analysed by microscopy and recorded. A slide was considered negative when no malaria parasite was seen in 100 highpower fields (HPF). A slide was considered positive when at least one parasite was seen in the thick film. All slides were double-read by different readers to ensure accuracy. Slides which were discrepant were excluded from the analysis.

### Cell reactive protein (CRP) test

CRP was measured using the CRP Test Kit CRP-K10 (Diagnostik Nord, Germany), a semi-quantitative membrane–based immunoassay for the determination of CRP in whole blood, serum or plasma. The test was standardized to detect CRP levels at or above 10 mg/l, with a reported sensitivity of 94.8% and a specificity of 98.3% (according to internal performance studies conducted by Diagnostik Nord). Between 10 and 12 drops of the buffer solution included in the test kit were put in a test tube, this was held vertically to prevent air trapping. A capillary tube was filled with approximately 3.5 μL of the patient`s serum and transferred into the tube filled with buffer solution and mixed thoroughly. Three drops of the specimen mixture were then put into the specimen well and the results were interpreted after 5 minutes. The appearance of a line in the “C” (control) marked zone confirmed that the test had worked properly. In the middle part of the test is the reference line “R”, with the test line “T” closest to the test field “S”. The results were then interpreted based on the coloured lines which appeared in the field lines. A test was said to be positive when three distinct colours appeared in the lines “T”, “C” and “R”. The CRP concentration of a sample is interpreted as 10–30 mg/l when the test line (T) is fainter than the reference line (R), around 30 mg/l when the test (T) is close to the reference line (R) in intensity and over 30 mg/l when the test line (T) is brighter than the reference line (R). The CRP concentration in the sample was less than 10 mg/l and the test was considered negative when two coloured lines appeared in the control (C) and reference (R) areas, with no distinct colour line appearing in the test area (T). A test was considered invalid when there was no control line (C) and/or reference line (R). This was then discarded and a new test was performed. [22].

### Data management and analysis

The association between the outcome variable parasitemia and possible impact variables were analysed performing a univariate analysis and then a multivariable logistic regression with backward stepwise selection. Parameters with potential relevance such as age, sex, tachycardia, tachypnea, blood culture result and white blood cell count were included in the final logistic regression model to assess their independent effect on parasitemia. For the stepwise estimation, a p-value of <0.1 was set as statistical cut-off meaning that variables with a p-value <0.1 were further analysed. Odds ratio (OR), 95% confidence interval (CI) and p-values were then calculated.

## Results

### Study population

Overall, 541 patients aged up to fifteen years were included in the study (Table 1). One patient had three and five patients had two visits, making up a total of 548 visits. The minimum age was one and the maximum was 167 months. The mean age was 40.5 months (SD ±37.7) and the median age 30 months (interquartile range 11–59). 419 (77.4%) of the children were ≤ 60 months old and 121 (22.6%) were > 60 months and ≤ 15 years old; 58.4% were male and 41.6% were female. Children < 12 months had much more often low CRP levels. The other basic parameters were similarly distributed over CRP levels.

**Table 1:** Basic parameters and distribution of CRP levels in 541 patients.

	Total	C-Reactive Protein			
	n = 541	low (<10 mg/l)	medium (10–30 mg/l)	high (>30 mg/l)	P
Sex (%)					0.642
Male	316 (58.4)	27 (64.3)	80 (59.7)	209 (57.3)	
Female	225 (41.6)	15 (35.7)	54 (40.3)	156 (42.7)	
Age, months (%)					<0.001
< 12	141 (26.1)	28 (66.7)	38 (28.4)	75 (20.6)	
12 - < 24	97 (17.9)	5 (11.9)	21 (15.7)	71 (19.5)	
24 - < 36	76 (14.1)	3 (7.1)	25 (18.7)	48 (13.2)	
36 - < 48	72 (13.3)	1 (2.4)	19 (14.2)	52 (14.3)	
48 - < 60	33 (6.1)	2 (4.8)	7 (5.2)	24 (6.6)	
≥ 60	122 (22.6)	3 (7.1)	24 (17.9)	95 (26.0)	
Ethnicity (%)					0.595
Akan	376 (69.5)	27 (64.3)	85 (63.4)	264 (72.3)	
Ewe	23 (4.3)	1 (2.4)	6 (4.5)	16 (4.4)	
Ga	2 (0.4)	0 (0)	1 (0.8)	1 (0.3)	
Northern	125 (23.1)	12 (28.6)	37 (27.6)	76 (20.8)	
Others	15 (2.8)	2 (4.8)	5 (3.7)	8 (2.2)	
House type (%)					0.855
Cement/brick	407 (75.2)	32 (76.2)	97 (72.4)	278 (76.2)	
Mud	133 (24.6)	10 (23.8)	37 (27.6)	86 (23.6)	
Wood	1 (0.2)	0 (0)	0 (0)	1 (0.3)	
Electricity (%)					0.778
Yes	325 (60.1)	27 (64.3)	82 (61.2)	216 (59.2)	
No	216 (39.9)	15 (35.7)	52 (38.8)	149 (40.8)	
Water source (%)					0.805
Inside Tap	10 (1.9)	0 (0)	4 (3.0)	6 (1.6)	
Stand pipe	438 (81.0)	35 (83.3)	106 (79.1)	297 (81.4)	
River water	46 (8.5)	3 (7.1)	14 (10.5)	29 (8.0)	
Well	47 (8.7)	4 (9.5)	10 (7.5)	33 (9.19)	

### Prevalence of *Plasmodium* parasitemia

Out of the 548 visits, thick blood smear results were available for all the patients. 270 (49.3%) blood smears yielded parasitemia with *Plasmodium* spp. and 278 (50.7%) were negative. Among patients above 12 months of age who were screened, 88.5% had a parasitemia, whereas among those aged up to 12 months, the prevalence was 11.5%. 76.6% of positive blood smears were found in children below 5 years of age.

### CRP levels, malarial parasitemia and septicemia

CRP levels were low (< 10 mg/l) in 42 (7.7%) visits and high (> 30 mg/l) in 368 (67.2%) visits. Few patients with negative CRP were malaria positive (7.1%), whereas more than half of patients with an increased CRP concentration (≥ 10 mg/l) were malaria positive (OR 14.5 [CI 4.4–47.6], p<0.001) (Table 2). Similarly, increased CRP levels were strongly associated with clinical malaria, defined as parasitemia > 5000 parasites/μl (OR 16.5 [CI 2.2–121], p<0.001).

**Table 2:** Univariate analysis of the association between CRP levels and parasitemia, malaria and septicemia in 548 patient visits.

CRP level	Parasitemia			Malaria			Septicemia*		
	No (%)	Yes (%)	OR (95% CI)	No (%)	Yes (%)	OR (95% CI)	No (%)	Yes (%)	OR (95% CI)
low (< 10 mg/l)	39 (92.9)	3 (7.1)	1	41 (97.6)	1 (2.4)	1	37 (92.5)	3 (7.5)	1
medium (10–30 mg/l)	66 (47.8)	72 (52.2)	14.2 (4.2–48.1)	105 (76.1)	33 (23.9)	12.9 (1.7–97.3)	114 (93.4)	8 (6.6)	0.9 (0.2–3.4)
high (> 30 mg/l)	173 (47.0)	195 (53.0)	14.7 (4.4–48.3)	256 (69.6)	112 (30.4)	17.9 (2.4–132)	323 (94.2)	20 (5.8)	0.8 (0.2–2.7)
increased (≥ 10 mg/l)	239 (47.2)	267 (52.8)	14.5 (4.4–47.6)	361 (71.3)	145 (28.7)	16.5 (2.2–121)	437 (94.0)	28 (6.0)	0.8 (0.2–2.7)

OR, odds ratio; CI, confidence interval

* n = 505, blood culture results not available for 43 patient visits

Blood culture results were available for 505 patients. Of them, 31 (6.1%) patients were positive for bacteria (Table 2). CRP levels were not associated with the likelihood of positive blood cultures (medium CRP vs. low CRP, OR 0.9 [CI 0.2–3.4], p = 0.84; high CRP vs. low CRP, OR 0.8 [CI 0.2–2.7], p = 0.68).

### Multivariate analysis

Patients with an increased CRP level had more than an eight-fold likelihood for positive parasitemia (adjusted OR 8.7 [CI 2.5–30.5], p<0.001) in the adjusted multivariable model (Table 3). Sex, tachycardia and tachypnea were not significantly associated with parasitemia. The adjusted OR of patients with a CRP > 10 mg/l for malaria was nine (adjusted OR 9.1 [CI 1.2–69.2], p = 0.034). After adjustment, increased CRP levels were not significantly associated with septicemia.

**Table 3:** Multivariate analysis of the association between CRP levels and other parameters, and parasitemia, malaria and septicemia in 548 patient visits.

Parameter	Parasitemia (n = 482)	Malaria (n = 482)	Septicemia (n = 441)
	OR (95% CI)	OR (95% CI)	OR (95% CI)
Basis	1	1	1
CRP ≥ 10 mg/l	8.7 (2.5–31.0)	9.2 (1.2–70.6)	0.8 (0.2–3.1)
Leukocytes [/μl]			
8,000 - < 16,000	0.6 (0.4–0.9)	0.7 (0.5–1.2)	1.1 (0.4–2.8)
≥ 16,000	0.2 (0.1–0.4)	0.2 (0.1–0.5)	1.3 (0.4–3.9)
Age [months]			
12 - < 24	4.0 (2.1–7.6)	3.3 (1.5–7.2)	0.8 (0.2–2.5)
24 - < 36	4.6 (2.3–9.0)	3.0 (1.3–6.5)	1.1 (0.3–3.7)
36 - < 48	6.4 (3.1–12.9)	3.8 (1.8–8.3)	0.2 (0.02–1.8)
48 - < 60	5.8 (2.4–14.1)	5.1 (2.0–13.1)	0.5 (0.05–3.9)
≥ 60	2.8 (1.5–5.3)	2.6 (1.2–5.7)	0.8 (0.2–2.6)
Sex female	1.4 (0.9–2.1)	1.3 (0.8–2.0)	2.1 (0.9–4.6)
Tachycardia	2.9 (1.2–7.2)	6.5 (1.4–29.1)	0.7 (0.1–3.6)
Tachypnea	0.5 (0.3–1.1)	0.4 (0.2–1.1)	0.7 (0.2–3.1)

OR, odds ratio; CI, confidence interval

Basis: CRP < 10 mg/l, leucocytes ≤ 8,000/μl, age < 1 years, sex male, no tachycardia, no tachypnea

### Sensitivity, specificity, positive and negative predictive values of CRP

In assessing the quality of the test, sensitivity, specificity, positive predictive value (PPV) and negative predictive value (NPV) of increased CRP for parasitemia, malaria and septicemia were calculated (Table 4). For parasitemia, these values were 98.9% (CI 96.8%-99.8%), 14.0% (CI 10.2%-18.7%), 52.8% (CI 48.3%-57.2%) and 92.9% (CI 80.5%-98.5%), respectively. Sensitivity, specificity, PPV and NPV of an increased CRP level for malaria were 99.3% (CI 96.2%-100%), 9.2% (CI 6.4%-12.8%), 31.7% (CI 27.4%-36.1%) and 97.0% (CI 84.2%-99.9%), respectively.

**Table 4:** Test quality of CRP levels for the prediction of parasitemia, malaria and septicemia.

	Parasitemia	Malaria	Septicemia
Sensitivity	98.9% (96.8%-99.8%)	99.3% (96.2%-100%)	90.3% (74.2%-98.0%)
Specificity	14.0% (10.2%-18.7%)	9.3% (6.4%-12.8%)	7.8% (5.6%-10.6%)
ROC area	0.57	0.54	0.50
Assumed prevalence	49%	30%	6.1%
PPV	52.8% (48.3%-57.2%)	31.7% (27.4%-36.1%)	6.0% (4.0%-8.6%)
NPV	92.9% (80.5%-98.5%)	97.0% (84.2%-99.9%)	92.5% (79.6%-98.4%)

ROC, Receiver-Operating Characteristic; PPV, positive predictive value; NPV, negative predictive value

## Discussion

The usefulness of CRP as a biomarker for the prediction of *Plasmodium* parasitemia was assessed in an area where malaria infection is very prevalent. The intention was to find out if CRP could help in distinguishing clinical malaria manifestations from other causes. As recommended by the WHO, all febrile patients should be tested for malaria and those who test positive for parasitemia should be treated for malaria. CRP must therefore not be considered as a replacement of proper parasitological diagnosis.

In the univariate analysis, elevated CRP levels were seen in patients with parasitemia and those with malaria. CRP was also associated to parasitemia in the multivariate analysis. Almost all of the patients with a detectable parasitemia (267/270, 98.9%) and those with clinical malaria (144/145, 99.3%) had elevated CRP levels. This finding is consistent with results from studies conducted in The Gambia, Tanzania, Mozambique and Malawi [4, 8, 16, 23] where malaria was also found to be associated with elevated CRP levels.

However, the positive predictive values of elevated CRP for parasitemia and malaria were only 52.8% and 31.7%, respectively. Accordingly, in patients with elevated CRP levels, about a half had no parasitemia and two-thirds had no malaria. This is similar to the background frequency of parasitemia and malaria found in the patients recruited for the study, indicating that there was no additional information provided by an increase of CRP levels in such an area.

CRP is known to rise during bacterial infections [16, 17]. In the present study, elevated CRP levels were found in 90.3% of the patients with positive blood culture results. The semi-quantitative test could not differentiate between parasitemia and bacterial infections.

Negative predictive values of elevated CRP were high for parasitemia (92.9%) and malaria (97.0%), implying that the likelihood that patients with normal CRP levels have no parasitemia or malaria is high. Based on this finding, a normal CRP may be used in ruling out malaria in febrile children in a context where malaria microscopy and RDT may not be readily available. However, CRP levels were normal only in a minority of patients.

In a recent study from Malawi the usefulness of different biomarkers was examined, namely C3a, C5a, angiopoientin-1, angiopoientin-2, soluble tie 2 (sTie-2), soluble Endoglin (sEndoglin), vascular endothelial growth factor (VEGF), soluble fms-like tyrosine kinase-1 (sFlt-1), tissue factor and leptin to identify plasmodium parasites in pregnant women. Individually, all these markers showed only a moderate ability (areas under ROC between 0.62 and 0.72) to identify malaria [8]. Similarly, CRP levels had a limited ability to identify malaria in the study presented here.

From our results, though CRP is elevated in parasitemia and in malaria, it lacks the ability to differentiate a *Plasmodium* spp. infection from septicemia due to its poor specificity. However, in a setting like sub-saharan Africa where other febrile illnesses play an important role, a negative CRP can nearly rule out parasitemia or malaria due to its high negative predictive value. The high negative predictive value results from the relative low frequency and cannot be generalised for situations with very high prevalence. Nevertheless, this finding could be helpful for clinicians in making decisions, especially in highly malaria-endemic areas, where fever is frequently equivalent to malaria and treated accordingly [24], leaving alternative agents of invasive infections unrecognized [25].

A limitation of this study was the use of only microscopy at 100 HPF to detect parasitemia without the use of PCR-diagnostics. As PCR is known to be more sensitive than microscopy examination [26], the use of micoroscopy alone might have led to some of the true cases of parasitemia being missed.

## Conclusion

The semi-quantitative method of measuring CRP is cheap, rapid and easy to perform, but not useful in predicting malaria or parasitemia in febrile children. Furthermore, it is unable to differentiate malaria from other febrile illnesses like septicemia due to its poor specificity. Nevertheless, a negative CRP has a high negative predictive value in few patients with normal CRP, meaning a negative CRP might be indicative of those patients unlikely to be presenting with clinical malaria. However, CRP cannot replace parasitological testing in febrile patients.

## Supporting information

S1 Data File CRP (XLS)
